# Role of Cholinergic Anti-Inflammatory Pathway in Treatment of Intestinal Ischemia-Reperfusion Injury by Electroacupuncture at Zusanli

**DOI:** 10.1155/2017/6471984

**Published:** 2017-12-03

**Authors:** Yanxia Geng, Dong Chen, Jiang Zhou, Hua Jiang, Haidong Zhang

**Affiliations:** ^1^Intensive Care Unit, Affiliated Hospital of Nanjing University of Traditional Chinese Medicine, 155 Hanzhong Road, Nanjing 210029, China; ^2^Acupuncture and Rehabilitation Department, Affiliated Hospital of Nanjing University of Traditional Chinese Medicine, 155 Hanzhong Road, Nanjing 210029, China

## Abstract

Electroacupuncture (EA) at Zusanli is a widely used method for the treatment of intestinal ischemic disease. The current study attempts to investigate the possible mechanism from the point of cholinergic anti-inflammatory pathway (CAP) in rats. Thirty rats were divided into five groups: control group, I/R group, EA group (I/R + EA), PNU group (I/R + *α*7 nAChR agonist), and *α*-BGT group (I/R + EA + *α*7 nAChR antagonist). EA and medicine injection were performed immediately after ischemia. After 2 h of reperfusion, blood and intestine samples were collected and intestinal histopathological score, mRNA expression of mucosal *α*7 nAChR and NF-*κ*Bp65, and serum cytokine levels (IL-6, TNF-*α*) were examined. Compared with the I/R group, the EA group and PNU group could significantly attenuate the mucosal damage, promote *α*7 nAChR mRNA expression, and reduce levels of NF-*κ*Bp65, IL-6, and TNF-*α*. Compared with the EA group, *α*7 nAChR mRNA was decreased, while concentrations of NF-*κ*Bp65, IL-6, and TNF-*α* increased in the *α*-BGT group. EA at Zusanli could inhibit NF-*κ*Bp65 and proinflammatory cytokines production after intestinal I/R injury; its mechanism may be related to the cholinergic anti-inflammatory pathway.

## 1. Background

Intestinal ischemic disease is a common clinical condition with limited treatment options. The Chinese medicine of electroacupuncture (EA) therapy has opened up a new field for this disease. Our previous study has shown that EA at Zusanli could reduce the expression of inflammatory cytokines, alleviate intestinal hyperpermeability, and promote repair after intestinal ischemia-reperfusion (I/R) injury [1]. However, the specific mechanism has not yet been clarified. In recent years, the cholinergic anti-inflammatory pathway (CAP) has been investigated a lot in the treatment of EA in intestinal inflammatory diseases [2], which might also help to illuminate the mechanism of ischemic disease, while ischemia is essentially an inflammatory process as well. The purpose of this study is to observe the effect of EA at Zusanli on the inflammatory response in rats' small intestine I/R injury and reveal its relationship with CAP, so as to provide a theoretical basis for clinical treatment.

## 2. Methods

### 2.1. Animal Grouping

 Intestinal I/R injury was carried out by superior mesenteric artery (SMA) occlusion for 30 min and release for 2 hours. Thirty male Sprague-Dawley rats weighing about 200–250 g were randomly divided into five groups: control group (Con), rats subjected to laparotomy with no clamping of SMA; I/R group, rats that underwent I/R injury; electroacupuncture group (EA), rats with I/R injury with EA applied (HANS/LH202H, 2 mA, 2–100 Hz) at bilateral Zusanli points (ST36 points) for 30 min, immediately after the ischemic period started; PNU282987 group (PNU), rats with I/R injury with PNU282987 (5 *μ*g/kg, Sigma) intraperitoneally injected immediately after ischemia started; *α*-bungarotoxin group (*α*-BGT), rats with I/R injury with *α*-bungarotoxin (1 *μ*g/kg, Sigma) intraperitoneally injected and EA applied simultaneously immediately after ischemia.

### 2.2. Histopathological Examination

Rats in each group were sacrificed at 2 h of reperfusion. 1 cm of the distal ileum was removed, rinsed, and fixed in 10% formaldehyde for H&E staining. Histological damage was assessed by Chiu's score under a light microscope.

### 2.3. RT-PCR Tests of *α*7 nAChR and NF-*κ*Bp65

Take 10 cm of the distal part of the small intestine, scrap off the mucosa, and grind it into powder in liquid nitrogen. Total RNA was extracted from the small intestine by TRIzol method (TransZol Up, TransGen). The transcription and amplification reaction were carried out according to the kit instructions (Thermo-Script RT-PCR System, Invitrogen). The primer sequences were as follows: *α*7 nAChR, 5′-ATCTGGGCATTGCCAGTATC-3′ (sense) and 5′-TCCCATGAGATCCCATTCTC-3′ (antisense); NF-кBp65, 5′-CACAGATACCACTAAGACGCACC-3′ (sense) and 5′-GACCGCATTCAAGTCATAGTCC-3′ (antisense); *β*-actin, 5′-TCAGGTCATCACTATCGGCAAT-3′ (sense) and 5′-AAAGAAAGGGTGTAAAACGCA-3′ (antisense). The expected length of the amplified fragments was 199 bp for *α*7 nAChR, 173 bp for NF-кBp65, and 432 bp for *β*-actin.

### 2.4. ELISA Tests of IL-6 and TNF-*α*

Collect 2 ml of blood from the portal vein and store the supernatant serum at −80°C after centrifugation. At the time of experiment, serum samples were thawed and ELISA tests of IL-6 and TNF-*α* were carried out according to the manufacturer's instructions (R&D Systems). Samples were incubated with biotin-labeled antibodies, streptavidin-HRP, substrates, and stop solution successively. The optical density (OD) value of each well was measured using a microplate reader. Standard curve was prepared according to the standard solution and corresponding OD value, and thus concentrations of IL-6 and TNF-*α* of each sample could be calculated.

### 2.5. Statistical Analysis

Data were analyzed by SPSS18.0 software. All values were expressed as mean ± standard deviation. One-way ANOVA was used to compare the data between groups. A *p* value of <0.05 was considered statistically significant.

## 3. Results

### 3.1. Histopathology Score of Small Intestine

As Table 1 shows, Chiu's score showed significantly more severe damage of the small intestine mucosa than that in the control group (3.7 ± 0.5 versus 0.3 ± 0.5, *p* < 0.01) after I/R injury. When compared with the I/R group, the histological destruction in EA and PNU groups was much relieved (*p* = 0.004 and 0.045, resp.). Chiu's score of the *α*-BGT group did not show a significant difference from the I/R group, but it was much higher than in the EA group (*p* = 0.014), indicating that *α*7 nAChR antagonist might reverse the efficacy of EA to some extent.

### 3.2. Mucosal mRNA Expression of *α*7 nAChR and NF-*κ*Bp65

**Figure 1:**
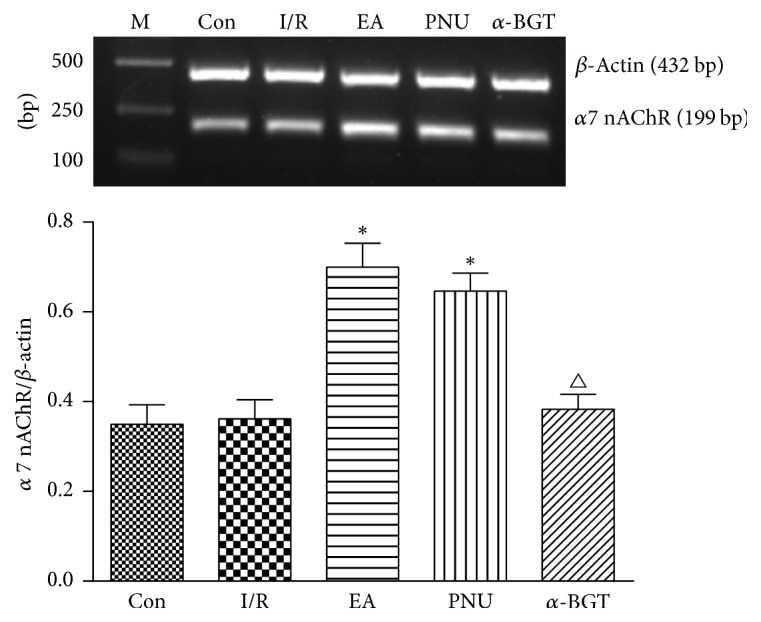
Mucosal *α*7 nAChR mRNA expression (blots and relative expressions) assessed by RT-PCR in each group. In the I/R group, mucosal concentration of *α*7 nAChR mRNA was similar to the control group and was significantly strengthened when applied with EA or PNU. In the *α*-BGT group, *α*7 nAChR mRNA level was much lower than in the EA group. M: marker; Con: control group; I/R: I/R group; EA: electroacupuncture group; PNU: PNU282987 group; *α*-BGT: *α*-bungarotoxin group; the abbreviations in Figure 2 are the same (^*∗*^*p* < 0.05 compared with the I/R group; ^△^*p* < 0.05 compared with the EA group).

The N-type cholinergic receptor (*α*7 nAChR) is the most studied receptor in the cholinergic anti-inflammatory pathway, and nuclear factor-*κ*B (NF-*κ*B) is the most important intracellular signal transduction pathway after *α*7 nAChR is activated. Our RT-PCR results showed that intestine mucosal expression of *α*7 nAChR mRNA in EA and PNU group was significantly strengthened compared with that in the I/R group, while in the *α*-BGT group, the difference was not that obvious. When compared with the EA group, *α*7 nAChR mRNA expression in the *α*-BGT group was significantly lower (Figure 1).

**Figure 2:**
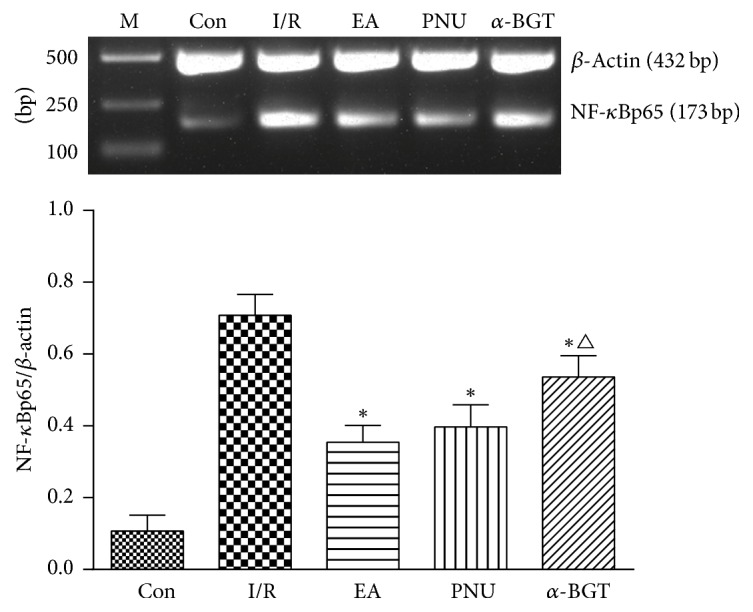
Mucosal NF-*κ*Bp65 mRNA expression (blots and relative expressions) assessed by RT-PCR in each group. Mucosal level of NF-*κ*Bp65 mRNA was significantly increased after I/R injury. After EA or PNU282987 was applied, the NF-*κ*Bp65 mRNA level was significantly decreased compared with the I/R group. Its concentration in the *α*-BGT group was higher than that in the EA group (^*∗*^*p* < 0.05 compared with the I/R group; ^△^*p* < 0.05 compared with the EA group).

The level of NF-*κ*Bp65 mRNA in intestine mucosa was remarkably elevated when suffering from I/R injury (*p* < 0.05). After EA or PNU282987 is applied, the NF-*κ*Bp65 mRNA expression was significantly decreased compared with the I/R group; nevertheless, the reduction was not so obvious in the *α*-BGT group (Figure 2).

### 3.3. Serum Inflammatory Factors Expression

**Figure 3:**
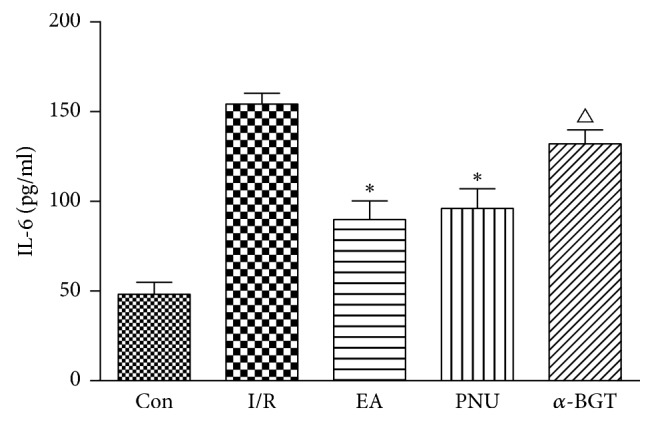
Serum IL-6 concentration in each group. Serum IL-6 concentration was significantly increased after I/R injury and was remarkably decreased in both EA and PNU groups when compared with the I/R group. In the *α*-BGT group, IL-6 level was higher than that in the EA group and showed no difference with the I/R group (^*∗*^*p* < 0.05 compared with the I/R group; ^△^*p* < 0.05 compared with the EA group).

**Figure 4:**
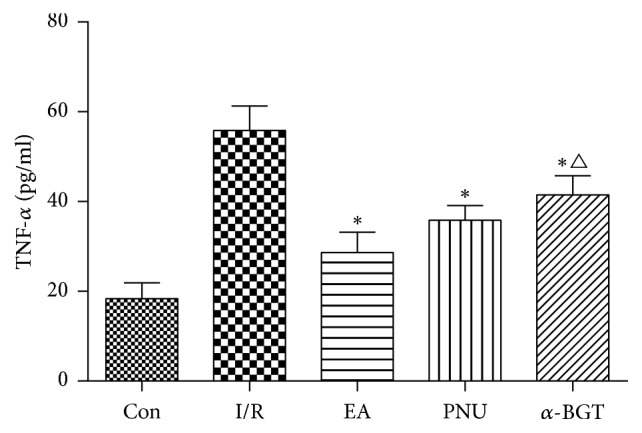
Serum TNF-*α* concentration in each group. Serum TNF-*α* level was greatly increased after I/R attack. Its concentration was significantly decreased in the three treatment groups compared with the I/R group. IL-6 level in the *α*-BGT group was higher than in the EA group (^*∗*^*p* < 0.05 compared with the I/R group; ^△^*p* < 0.05 compared with the EA group).

After intestinal I/R injury, serum levels of IL-6 and TNF-*α* were significantly increased compared with the control group (154.3 ± 14.4 versus 48.3 ± 16.0 for IL-6 and 55.8 ± 13.4 versus 18.3 ± 8.8 for TNF-*α*, resp.). Their concentrations were significantly downregulated in both EA and PNU groups (*p* < 0.01). Besides, the level of TNF-*α* in the *α*-BGT group was much lower than in the I/R group (*p* = 0.026) and was higher than that in the EA group (*p* = 0.044). Nevertheless, serum IL-6 in the *α*-BGT group was much higher than in the EA group (*p* = 0.002) but was not significantly different from that in the I/R group (Figures 3 and 4).

## 4. Discussion

The immunoregulation and anti-inflammatory effect of acupuncture therapy have been widely recognized. However, the understanding of its structure, function, and mechanism is not yet in depth. In recent years, activation of the nerve-immune regulation of the cholinergic anti-inflammatory pathway has gradually become a hotspot. In this theory, the therapeutic effects of acupuncture on internal organs may be mediated by vagal modulation of inflammatory responses [3]. That is, acupuncture can stimulate the vagus nerve and inhibit proinflammatory cytokines expression via interaction of neurotransmitters, acetylcholine, and *α*7nAChR subunit on reticuloendothelial macrophage cells [4].

Previous studies have shown that acupuncture at Zusanli could attenuate the systemic inflammatory response, protect intestinal barrier integrity, and improve organ function and survival rate in hemorrhaged rats [4]. EA at Zusanli also has the potential to reduce serum TNF level in septic rats, protect intestinal barrier integrity from ischemia injury, and reduce postoperative local inflammatory response to alleviate adhesion formation in rats [5–9]. Moreover, abdominal vagotomy or *α*7 nAChR inhibitor could reverse these protective effects of EA [4–9]. PNU-282987 is a selective *α*7 nAChR agonist. A previous study showed that pretreatment with PNU-282987 could prevent NF-*κ*B activation and suppress cytokine production after hepatic I/R injury in mice [10]. Our study also showed that PNU-282987 and EA have a similar effect in the inhibition of NF-*κ*B and cytokine expression after intestinal I/R injury in rats. These findings indicate that vagal nerve integrity and *α*7 nAChR subunit play an important role in its anti-inflammatory and organ protective effects.

NF-*κ*B is a common pathway regulating the transcription of various inflammatory factors. It plays a central role in sepsis and autoimmune diseases. In resting state, NF-*κ*B binds to NF-*κ*B inhibitory protein (I*κ*B) to form a trimer complex. When the cells are stimulated by external factors (such as cytokines and mediators), activated I*κ*B kinase causes ubiquitination and degradation of I*κ*B, thereby releasing NF-*κ*B from the trimer complex and transferring it to the nucleus to activate the transcription process. NF-*κ*B activation could lead to the expression of many genes, and its overactivation will inevitably cause a series of pathological reactions. Qin et al. reported that EA could suppress the activity of NF-*κ*B signaling pathway to ameliorate inflammatory injury in cerebral I/R rats [11]. It is also believed that NF-*κ*B is one of the main intracellular pathways after *α*7 nAChR activation, which can inhibit the phosphorylation of I*κ*B and nuclear translocation of NF-*κ*B and ultimately suppress proinflammatory cytokine production induced by endotoxin and trauma [12]. Studies have shown that nicotine can inhibit the lipopolysaccharide-induced I*κ*B degradation and NF-*κ*B nuclear translocation, thereby preventing TNF-*α* and HMBG1 production [13]. In our research, the content of NF-*κ*B mRNA in intestinal mucosa was significantly increased after I/R injury, and this increase was obviously alleviated after EA or *α*7 nAChR agonist was applied, which was consistent with previous findings.

Acupuncture is a convenient and effective method for gastrointestinal dysfunction diseases [14]. Electroacupuncture is a combination of acupuncture practices and electrophysiological effects. It can increase the needling sensation, produce stronger stimulation intensity, and reduce the work of the twirling needle and is easier to repeat than conventional acupuncture. Hence, it is now widely used in both research and clinical settings. The waveform of EA we performed in this study is condensation-rarefaction wave which is comprised of continuous alternation of condensation wave and rarefaction wave. It is not easy to produce adaption unlike single-pulse wave, and it is often used in situations attempting to increase metabolism, promote blood circulation, and eliminate inflammatory edema. Torres-Rosas et al. even found that the anti-inflammatory effect of EA at ST36 presents a voltage-dependent manner in lipopolysaccharide treated mice [15]. Grech et al. suggest that low-frequency EA can improve immune and stress responses to surgery in anesthetized patients [16]. Future studies can focus on whether different frequencies or voltages have different anti-inflammatory effects.

In a previous study, we have confirmed the anti-inflammatory and mucosal barrier protection effect of EA at Zusanli during intestinal I/R injury [1]. This study just attempts to explore its possible mechanism from the perspective of cholinergic anti-inflammatory pathway, and the results showed that both EA and *α*7 nAChR agonists could relieve the intestine damage and decrease levels of mucosal NF-*κ*Bp65 and serum IL-6 and TNF-*α*. Intraperitoneal administration of *α*7nAChR antagonist significantly eliminated the anti-inflammatory effect of EA. These observations suggest that EA at Zusanli could protect the intestine from I/R injury possibly by activating the cholinergic anti-inflammatory pathway through *α*7 nAChR subunit and NF-*κ*B intracellular signal transduction mechanism. Recently, Dhawan et al. showed that M-type cholinergic receptor (mAChR) also provides protective effects against colitis in in vitro experiments and that mAChR stimulation might block the phosphorylation of myosin light chain, inhibit myosin contraction, and thus maintain the tight junction protein in a close state and protect the epithelium barrier function [17, 18]. Therefore, more researches on this mechanism are needed to provide a complete theoretical basis for clinical application of Chinese medicine in intestinal ischemic situations.

## Figures and Tables

**Table 1:** Chiu's scoring in each group.

Groups	Chiu's score
Control	0.3 ± 0.5
I/R group	3.7 ± 0.5
EA group	2.7 ± 0.5^*∗*^
PNU group	3.0 ± 0.6^*∗*^
*α*-BGT group	3.5 ± 0.6^△^

^*∗*^ *p* < 0.05 compared with the I/R group; ^△^*p* < 0.05 compared with the EA group.
